# Racial disparities in the prevalence and control of hypertension among a cohort of HIV-infected patients in the southeastern United States

**DOI:** 10.1371/journal.pone.0194940

**Published:** 2018-03-29

**Authors:** Greer A. Burkholder, Ashutosh R. Tamhane, Monika M. Safford, Paul M. Muntner, Amanda L. Willig, James H. Willig, James L. Raper, Michael S. Saag, Michael J. Mugavero

**Affiliations:** 1 Division of Infectious Diseases, University of Alabama at Birmingham, Birmingham, Alabama, United States of America; 2 Department of Biostatistics, University of Alabama at Birmingham, Birmingham, Alabama, United States of America; 3 Division of General Internal Medicine, Weill Cornell Medical College, New York City, New York, United States of America; 4 Department of Epidemiology, University of Alabama at Birmingham, Birmingham, Alabama, United States of America; Katholieke Universiteit Leuven Rega Institute for Medical Research, BELGIUM

## Abstract

**Background:**

African Americans are disproportionately affected by both HIV and hypertension. Failure to modify risk factors for cardiovascular disease and chronic kidney disease such as hypertension among HIV-infected patients may attenuate the benefits conferred by combination antiretroviral therapy. In the general population, African Americans with hypertension are less likely to have controlled blood pressure than whites. However, racial differences in blood pressure control among HIV-infected patients are not well studied.

**Methods:**

We conducted a cross-sectional study evaluating racial differences in hypertension prevalence, treatment, and control among 1,664 patients attending the University of Alabama at Birmingham HIV Clinic in 2013. Multivariable analyses were performed to calculate prevalence ratios (PR) with 95% confidence intervals (CI) as the measure of association between race and hypertension prevalence and control while adjusting for other covariates.

**Results:**

The mean age of patients was 47 years, 77% were male and 54% African-American. The prevalence of hypertension was higher among African Americans compared with whites (49% vs. 43%; p = 0.02). Among those with hypertension, 91% of African Americans and 93% of whites were treated (p = 0.43). Among those treated, 50% of African Americans versus 60% of whites had controlled blood pressure (systolic blood pressure <140 mmHg and diastolic blood pressure <90 mmHg) (p = 0.007). After multivariable adjustment for potential confounders, prevalence of hypertension was higher among African Americans compared to whites (PR 1.25; 95% CI 1.12–1.39) and prevalence of BP control was lower (PR 0.80; 95% CI 0.69–0.93).

**Conclusions:**

Despite comparable levels of hypertension treatment, African Americans in our HIV cohort were less likely to achieve blood pressure control. This may place them at increased risk for adverse outcomes that disproportionately impact HIV-infected patients, such as cardiovascular disease and chronic kidney disease, and thus attenuate the benefits conferred by combination antiretroviral therapy.

## Introduction

Due to the effectiveness of combination antiretroviral therapy (ART), non-human immunodeficiency virus (HIV)-related diseases have become the predominant cause of morbidity and mortality among HIV-infected patients in high income countries [1]. In addition, a complex interplay of traditional risk factors, chronic inflammation and immune activation related to HIV, and antiretroviral toxicity place HIV-infected patients at increased risk for myocardial infarction, stroke, and chronic kidney disease (CKD) compared with their uninfected counterparts [2–4]. Hypertension is a modifiable risk factor for these outcomes and is common among HIV-infected patients [5–9]. Hypertension in HIV-infected patients is associated with an increased risk for MI, stroke, CKD, all-cause hospitalization, and mortality [10–14].

In the United States (US), it is well-recognized that the prevalence of hypertension is higher among African Americans compared with other racial/ethnic groups [15], and this finding has also been reported within HIV cohorts including our own [16–18]. Prior studies of the general population have reported that African Americans with hypertension are more likely to have uncontrolled blood pressure (BP) than whites despite similar rates of awareness and treatment [19, 20]. These disparities in hypertension prevalence and control contribute to higher rates of stroke, CKD, and congestive heart failure observed among African Americans compared to whites [21].

Failure to modify risk factors for cardiovascular disease (CVD) and CKD such as hypertension among HIV-infected patients may attenuate the benefits conferred by combination ART. From a public health policy perspective, information regarding BP control among HIV-infected patients and whether there are racial disparities which need to be addressed in this population are important. However, there are few published studies evaluating the association between race and BP control among HIV-infected patients.

The University of Alabama at Birmingham (UAB) 1917 HIV Clinic Cohort is located in the southeastern US, the epicenter of the contemporary American HIV epidemic [22]. In addition, the southeastern US has high rates of hypertension-related diseases (CVD and CKD) [19, 23, 24]. A disproportionate impact of HIV and hypertension-related diseases in this region are notable among African Americans, making it a pertinent setting for study of the intersection of these two disease states in this population. We conducted a cross-sectional study in the UAB 1917 HIV Clinic Cohort evaluating the association of race, among other patient characteristics, with the prevalence, treatment, and control of hypertension.

## Materials and methods

### Study design and setting

This cross-sectional study was nested within the UAB 1917 Clinic Cohort, a prospective HIV clinical cohort established in 1992 (http://www.uab.edu/medicine/1917cliniccohort/). The cohort’s electronic database contains extensive sociodemographic, clinical, and psychosocial information on patients receiving outpatient primary HIV and subspecialty care at the UAB 1917 HIV/AIDS Clinic (1917 Clinic). This study was approved by the UAB Institutional Review Board.

### Eligibility criteria

The study population included 1917 Clinic patients who had established care for at least one year as of December 31, 2013. In order to capture this population, the inclusion criteria were: 1) adults ≥ age 19 years; 2) at least one routine (non-urgent) HIV primary care visit in 2013; and 3) at least one routine HIV primary care visit in the period 12–24 months prior to the most recent visit in 2013. The focus on established patients (N = 1,717) allowed for a sufficient observation period to determine which patients had hypertension, as well as ample time for providers to initiate or intensify antihypertensive therapy to achieve guideline endorsed goals of treatment [25]. Patients who died on or prior to December 31, 2013 were excluded (n = 18). Patients of race/ethnicity other than African-American or non-Hispanic white were excluded due to low numbers (n = 35; including 20 Hispanic patients, 4 Asian, 4 multiracial, and 7 of unknown race/ethnicity).

**Fig 1:**
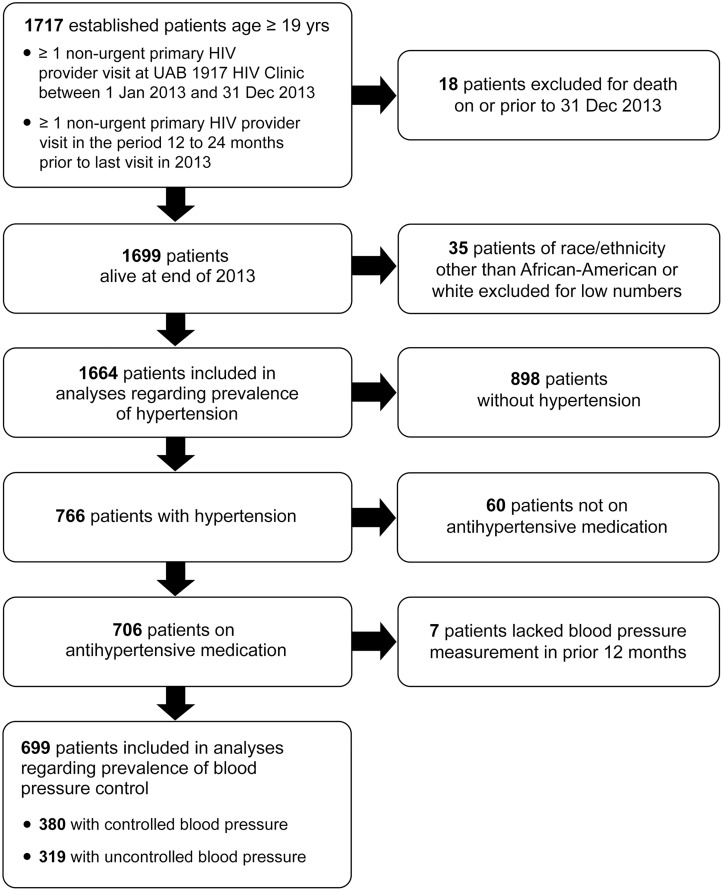
Flow diagram of criteria for inclusion in analyses of hypertension prevalence, treatment, and control among HIV-infected patients attending the UAB 1917 HIV Clinic in 2013.

After these exclusions were applied, data for 1,664 patients were available for the analyses of hypertension prevalence (Fig 1). The index visit for each patient was the last visit occurring between January 1 and December 31, 2013. For the analysis of antihypertensive treatment, the population included 766 patients with hypertension. For the BP control analyses, the population was further restricted to participants taking antihypertensive medication (n = 706) and an additional 7 patients lacking a documented BP in the 12 months prior to their index visit were excluded, resulting in 699 included patients.

### Sources of data

Sociodemographic data, comorbidities, medications, vital signs, laboratory results, and visit data were obtained by query of the cohort’s electronic database using MS SQL Server 2008. Psychosocial variables were obtained from our electronic Patient Reported Outcome (PRO) measures, validated instruments which are self-administered by patients at approximately 4–6 month intervals. These included tobacco use, alcohol use (Alcohol Use Disorders Identification Test-Consumption [AUDIT-C]), substance use (Alcohol, Smoking, and Substance Involvement Screening Test [ASSIST]), depression (Patient Health Questionnaire-9 [PHQ-9]), and antiretroviral adherence (Adult AIDS Clinical Trials Group [AACTG] adherence instrument) [26]. Data were deidentified prior to analyses.

### Primary outcomes

The primary outcomes were prevalence, treatment, and control of hypertension. Patients were categorized as having hypertension by meeting at least two of the following three criteria: 1) diagnosis of hypertension on the patient’s active problem list in the UAB electronic health record (EHR); 2) systolic blood pressure (SBP) ≥140 mmHg or diastolic blood pressure (DBP) ≥90 mmHg on two visits at least 7 days apart within 12 months prior to the index visit; 3) antihypertensive medication on the patient’s active medication list in the UAB EHR. Treatment of hypertension was defined by the presence of an antihypertensive medication on a patient’s active medication list. Control of hypertension was defined as SBP <140 mmHg and DBP <90 mmHg at the index visit [25].

Antihypertensive medication classes included angiotensin-converting enzyme inhibitors, angiotensin II receptor blockers, diuretics (with thiazide, loop, and potassium-sparing diuretics considered as separate classes), beta blockers, calcium channel blockers, alpha blockers, centrally acting agents, renin inhibitors, and direct vasodilators. Routine BP measurements taken during visits to the 1917 Clinic and other outpatient clinics within the UAB health system were used. These are obtained by trained clinic personnel, usually as a single measurement with an automated sphygmomanometer. If more than one BP measurement was reported for a visit, the average was used.

### Independent variables

Sociodemographic characteristics included race, sex, age, HIV transmission risk factor, and insurance status. Age was categorized as < 40, 40–59, or ≥60 years; HIV transmission risk factor as men who have sex with men, heterosexual, or intravenous drug use; and insurance status as private, public, or none.

Clinical characteristics included body mass index (BMI), CKD, diabetes mellitus (DM), history of CVD, CD4 count, plasma HIV-1 RNA (viral load [VL]), whether on ART, duration of ART, length of time in care at the 1917 Clinic, visit frequency in the prior 12 months, antihypertensive treatment intensity and frequency, and duration of antihypertensive medication use. BMI was categorized as obese (≥30 kg/m^2^), overweight (25–29.9 kg/m^2^), or normal/underweight (<25 kg/m^2^). CKD was defined as two estimated glomerular filtration rate measurements <60 mL/min/1.73m^2^ ≥90 days apart calculated using the Chronic Kidney Disease Epidemiology Collaboration equation or one spot urine albumin/creatinine ≥30mg/g in the 12 months prior to the index visit [27].

DM was defined as meeting at least two of the three following criteria: 1) DM diagnosis on a patient’s active problem list; 2) oral hypoglycemic therapy or insulin on a patient’s active medication list; or 3) at least one hemoglobin A1C value ≥6.5% within 12 months prior to the index visit. History of CVD was defined as ever having a diagnosis of stroke or cerebrovascular disease, transient ischemic attack, coronary heart disease, MI, unstable angina, or angina pectoris or documentation of a coronary revascularization procedure in the UAB EHR.

CD4 count was categorized as <200 versus ≥200 cells/μL and VL as <200 versus ≥ 200 copies/mL. Antihypertensive treatment intensity was categorized as 1, 2, or ≥3 classes of medication and antihypertensive medication frequency as once daily versus more than once daily.

Psychosocial characteristics included current tobacco use, at risk alcohol use, substance use, depressive symptoms, and adherence to ART. At risk alcohol use was defined by an AUDIT-C score of ≥4 in women and ≥5 in men; substance use by current use of cocaine, amphetamines, non-prescribed opioids, or intravenous drugs as per the ASSIST; depressive symptoms as “major depression” or “other depression” per PHQ-9 scoring algorithm; and ART adherence as no missed doses within 2 weeks prior to completing the AACTG adherence instrument [26]. For time-varying covariates, the nearest value on or prior to the index visit was used. If no value was available within 12 months prior to the index visit, it was considered missing.

### Statistical analysis

The prevalence, treatment, and control of hypertension were calculated for the overall population and for African Americans versus whites. Characteristics of patients were calculated for the overall population, and stratified by race, the presence of hypertension, and BP control status. Continuous variables are reported as means with standard deviation or medians with first and third quartiles, and categorical variables as frequencies with percentages.

Univariate and multivariable analyses were performed to calculate prevalence ratios (PR) with 95% confidence intervals (CI) as the measure of association between race and hypertension prevalence while adjusting for other covariates. For multivariable modeling, due to convergence problems in log binomial regression, modified Poisson regression with robust error variance was used [28]. Variables were selected for the multivariable model based on clinical relevance (race, age, sex, BMI, CKD, DM, smoking, alcohol abuse, and ART duration) with additional variables included based on a moderate univariate association (PR <0.80 or >1.20) with hypertension (insurance status, CD4, VL). Univariate and multivariable log binomial regression modeling was performed to evaluate the association between race and controlled BP while adjusting for other covariates among patients treated for hypertension. Duration of antihypertensive medication use, number of antihypertensive classes, and antihypertensive medication dosing frequency were included in the multivariable model, in addition to the same clinically relevant variables used in the hypertension prevalence model. Multi-collinearity of the independent variables was examined with variance inflation factor (VIF) by adjusting the linear combinations by the weight matrix used in the maximum likelihood algorithm; the VIF for all the factors was <2.6 thus indicating no multi-collinearity [29]. Statistical significance was set at p = 0.05 (two-sided). Analyses were performed using SAS statistical software (Cary, North Carolina), version 9.3.

## Results

### Participant characteristics

Among the 1,664 patients meeting study eligibility criteria, the mean age was 47 years (standard deviation (SD): 11 years), 77% were male and 54% African-American (Table 1). African Americans were younger than whites (mean age 45 vs. 48 years), and a higher proportion were female (32% vs. 12%), uninsured (32% vs. 24%), and obese (35% vs. 20%) (all p <0.001). Prevalence of CVD was lower among African Americans than whites (6% vs. 9%; p = 0.008), while prevalence of CKD and DM were similar. A similar proportion of African Americans and whites were treated with ART (96% vs. 98%; p = 0.66), however viral suppression on ART was less common among African Americans (86% vs. 94%, p<0.001). Among patients completing the AACTG adherence instrument, African Americans were less likely to report adherence to ART than whites (73% vs. 81%; p<0.001).

**Table 1:** Characteristics of HIV-infected patients attending the UAB 1917 HIV Clinic in 2013 included in the analyses of hypertension prevalence.

Characteristic	Overall population	African-American	White	p-value^c^
	n (%) or median (IQR)^b^	n (%)^a^ or median (IQR)^b^	n (%)^a^ or median (IQR)^b^	
	(N = 1664)	(n = 894)	(n = 770)	
**Age, years**	46.6 (10.9)^d^	45.1 (11.4)^d^	48.4 (10.0)^d^	<0.001
**Age, categorical, years**				<0.001
<40	426 (25.6)	289 (32.3)	137 (17.8)	
40–59	1065 (64.0)	517 (57.8)	548 (71.2)	
≥60	173 (10.4)	88 (9.9)	85 (11.0)	
**Sex**				<0.001
Female	380 (22.8)	288 (32.2)	92 (12.0)	
Male	1284 (77.2)	606 (67.8)	678 (88.0)	
**HIV transmission risk factor**				<0.001
Heterosexual	584 (35.8)	442 (50.7)	142 (18.6)	
MSM	905 (55.4)	378 (43.4)	527 (69.2)	
IVDU	144 (8.8)	51 (5.9)	93 (12.2)	
**Insurance status**				<0.001
Private	649 (39.0)	306 (34.2)	343 (44.6)	
Public	541 (32.5)	302 (33.8)	239 (31.0)	
None	474 (28.5)	286 (32.0)	188 (24.4)	
**BMI, kg/m**^**2**^				<0.001
Normal/Underweight (<25)	655 (39.4)	302 (33.8)	353 (45.8)	
Overweight (25–29.9)	546 (32.8)	281 (31.4)	265 (34.4)	
Obese (≥30)	463 (27.8)	311 (34.8)	152 (19.8)	
**Chronic kidney disease**				0.40
No	1484 (89.8)	792 (89.2)	692 (90.5)	
Yes	169 (10.2)	96 (10.8)	73 (9.5)	
**Diabetes mellitus**				0.31
No	1512 (90.9)	806 (90.2)	706 (91.7)	
Yes	152 (9.1)	88 (9.8)	64 (8.3)	
**History of CVD**		)		0.008
No	1542 (92.7)	843 (94.3)	699 (90.8)	
Yes	122 (7.3)	51 (5.7)	71 (9.2)	
**On ART**				0.12
No	51 (3.1)	33 (3.7	18 (2.3)	
Yes	1613 (96.9)	861 (96.3)	752 (97.7)	
**Duration of ART, years**	6.3 (3.2–11.4)	5.4 (2.9–9.9)	7.5 (3.7–13.0)	<0.001
**CD4 count, cells/μL**				0.004
≥200	1530 (92.3)	807 (90.6)	723 (94.4)	
<200	127 (7.8)	84 (9.4)	43 (5.6)	
**VL, copies/mL for all patients**				<0.001
≥200	196 (11.8)	139 (15.6)	57 (7.4)	
<200	1458 (88.2)	750 (84.4)	708 (92.6)	
**VL, copies/mL for patients on ART**				<0.001
≥200	171 (10.7)	123 (14.4)	48 (6.4)	
<200	1433 (89.3)	733 (85.6)	700 (93.6)	
**Time in care at HIV clinic, years**	6.6 (3.5–12.3)	5.7 (3.0–10.9)	8.0 (4.1–13.4)	<0.001
**Cumulative visits in last 12 months**	3 (2–4)	3 (2–4)	3 (2–3)	<0.001
**Adherent to ART**				<0.001
No	267 (16.1)	161 (18.0)	106 (13.8)	
Yes	909 (54.6)	443 (49.6)	466 (60.5)	
Missing	488 (29.3)	290 (32.4)	198 (25.7)	
**Smoking**				<0.001
Never	538 (32.3)	342 (38.3)	196 (25.5)	
Prior	298 (17.9)	117 (13.1)	181 (23.5)	
Current	407 (24.5)	193 (21.5)	214 (27.8)	
Missing	421 (25.3)	242 (27.1)	179 (23.2)	
**At risk alcohol use**				<0.001
No	983 (59.1)	532 (59.5)	451 (58.6)	
Yes	248 (14.9)	107 (12.0)	141 (18.3)	
Missing	433 (26.0)	255 (28.5)	178 (23.1)	
**Current substance abuse**				0.05
No	1125 (67.6)	582 (65.1)	543 (70.5)	
Yes	88 (5.3)	54 (6.0)	34 (4.4)	
Missing	451 (27.1)	258 (28.9)	193 (25.1)	
**Current depression**				0.08
No	1075 (64.6)	559 (62.5)	516 (67.0)	
Yes	211 (12.7)	113 (12.7)	98 (12.7)	
Missing	378 (22.7)	222 (24.8)	156 (20.3)	

^a^Column percents.

^b^Median with first and third quartiles

^c^ p-values calculated using Pearson chi-square test (proportions), t-test (means), and Wilcoxon rank-sum test (medians)

^d^Mean with standard deviation.

Abbreviations: ART, antiretroviral therapy; BMI, body mass index; CVD, cardiovascular disease; HIV, human immunodeficiency virus; IQR, interquartile range; IVDU, intravenous drug use; MSM, men who have sex with men; UAB, University of Alabama at Birmingham; VL, HIV viral load.

Missing data: HIV transmission risk factor, 30 (plus 1 patient excluded for risk factor of hemophilia/blood transfusion); chronic kidney disease, 11; CD4 cell count, 7; VL, 10.

### Hypertension prevalence, treatment, and control

**Fig 2:**
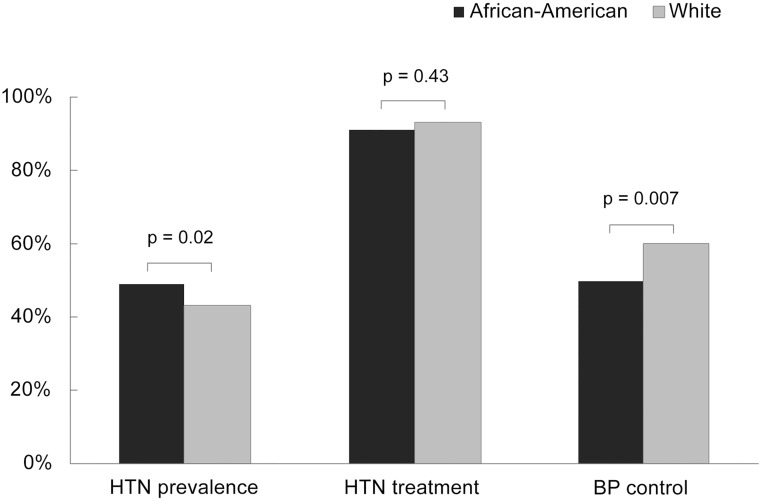
Prevalence, treatment, and control of hypertension stratified by race among HIV-infected patients attending the UAB 1917 HIV Clinic in 2013.

Overall, 46% of patients had hypertension with a higher prevalence among African Americans compared with whites (49% vs. 43%; p = 0.02) (Fig 2). Of those with hypertension, 91% of African Americans and 93% of whites were taking antihypertensive medication (p = 0.43). Among all patients with hypertension, 51% had controlled BP (SBP/DBP <140/90 mmHg), and among those taking antihypertensive medications, 54% had controlled BP. African Americans taking antihypertensive medications were less likely than whites to have controlled BP (50% vs. 60%; p = 0.007). Among those with uncontrolled BP, African Americans were more likely than whites to have SBP ≥160 mmHg and/or DBP ≥100 mmHg (36% vs. 23%; p = 0.02). Among patients treated for hypertension, the median duration of antihypertensive medication use was 4.8 (IQR: 2.5–8.3) years among African Americans and 5.3 (IQR: 3.1–8.7) years among whites (p = 0.18). A similar proportion of African Americans and whites were on antihypertensive regimens taken once daily (66% vs. 67%), however African-Americans were more likely to be on ≥3 classes of antihypertensive medications than whites (28% vs. 18%; p = 0.003).

### Patient characteristics associated with hypertension prevalence

African American race was associated with a higher prevalence of hypertension in univariate (PR 1.13; 95% CI 1.02–1.26) and multivariable (PR 1.25; 95% CI 1.12–1.39) analyses compared to whites (Table 2). Other patient characteristics associated with higher prevalence of hypertension in multivariable analysis included older age, being overweight or obese, CKD, DM, history of CVD, and longer duration of ART. Current substance use was associated with lower prevalence of hypertension.

**Table 2:** Characteristics associated with prevalence of hypertension among 1,664 HIV-infected patients attending the UAB 1917 HIV Clinic in 2013.

Characteristic	Hypertension	No Hypertension	Univariate analysis		Multivariable analysis^c^	
	(n = 766)	(n = 898)				
	n (%)^a^ or	n (%)^a^ or	Prevalence ratio	p-value	Prevalence ratio	p-value
	median (IQR)^b^	median (IQR)^b^	(95%CI)		(95%CI)	
**Age, years**						
<40	90 (21.1)	336 (78.9)	1.00	-	1.00	-
40–59	543 (51.0)	522 (49.0)	2.41 (1.99–2.93)	<0.001	2.08 (1.70–2.53)	<0.001
≥60	133 (76.9)	40 (23.1)	3.64 (2.98–4.45)	<0.001	2.76 (2.21–3.45)	<0.001
**Sex**						
Female	200 (52.6)	180 (47.4)	1.00	-	1.00	-
Male	566 (44.1)	718 (55.9)	0.84 (0.75–0.94)	0.002	1.03 (0.92–1.15)	0.63
**Race**						
White	331 (43.0)	439 (57.0)	1.00	-	1.00	-
African American	435 (48.7)	459 (51.3)	1.13 (1.02–1.26)	0.02	1.25 (1.12–1.39)	<0.001
**HIV transmission risk factor**						
Heterosexual	307 (52.6)	277 (47.4)	1.00	-	-	-
MSM	385 (42.5)	520 (57.5)	0.81 (0.73–0.90)	0.001	-	-
IVDU	63 (43.7)	81 (56.3)	0.83 (0.68–1.02)	0.07	-	-
**Insurance status**						
Private	330 (50.8)	319 (49.2)	1.00	-	1.00	-
Public	275 (50.8)	266 (49.2)	1.00 (0.89–1.12)	1.00	0.97 (0.87–1.09)	0.62
None	161 (34.0)	313 (66.0)	0.67 (0.58–0.77)	<0.001	0.88 (0.77–1.02)	0.08
**BMI, kg/m**^**2**^						
Normal/Underweight (<25)	231 (35.3)	424 (64.7)	1.00	-	1.00	-
Overweight (25–29.9)	259 (47.4)	287 (52.6)	1.34 (1.17–1.54)	<0.001	1.23 (1.08–1.40)	0.002
Obese (≥30)	276 (59.6)	187 (40.4)	1.69 (1.48–1.92)	<0.001	1.54 (1.35–1.76)	<0.001
**Chronic kidney disease**						
No	636 (42.9)	848 (57.1)	1.00	-	1.00	-
Yes	124 (73.4)	45 (26.6)	1.71 (1.54–1.91)	<0.001	1.23 (1.09–1.39)	<0.001
**Diabetes mellitus**						
No	645 (42.7)	867 (57.3)	1.00	-	1.00	-
Yes	121 (79.6)	31 (20.4)	1.87 (1.69–2.06)	<0.001	1.29 (1.15–1.45)	<0.001
**History of CVD**						
No	680 (44.1)	862 (55.9)	1.00	-	1.00	-
Yes	86 (70.5)	36 (29.5)	1.60 (1.41–1.82)	<0.001	1.26 (1.10–1.44)	<0.001
**Duration of ART, years**	8.2 (4.2–13.1)	5.1 (2.7–9.6)	1.18 (1.13–1.22)^d^	<0.001	1.09 (1.04–1.14)^d^	<0.001
**CD4 count, cells/μL**						
≥200	720 (47.1)	810 (52.9)	1.00	-	1.00	-
<200	44 (34.7)	83 (65.3)	0.74 (0.58–0.94)	0.01	0.83 (0.65–1.07)	0.16
**HIV viral load, copies/mL**						
≥200	64 (32.6)	132 (67.4)	1.00	-	1.00	-
<200	698 (47.9)	760 (52.1)	1.46 (1.19–1.80)	<0.001	1.20 (0.99–1.46)	0.07
**Time in care at HIV clinic, years**	8.3 (4.3–13.6)	5.5 (2.8–10.6)	1.05 (1.04–1.06)^d^	<0.001	-	-
**Cumulative visits in last 12 months**	3 (2–4)	3 (2–3)	1.03 (1.00–1.07)^d^	0.03	-	-
**Current smoking**						
No	410 (49.0)	426 (51.0)	1.00	-	1.00	-
Yes	160 (39.3)	247 (60.7)	0.80 (0.70–0.92)	0.002	0.95 (0.83–1.09)	0.47
Missing	196 (46.6)	225 (53.4)	0.95 (0.84–1.07)	0.41	0.87 (0.67–1.15)	0.33
**At risk alcohol use**						
No	456 (46.4)	527 (53.6)	1.00	-	1.00	-
Yes	106 (42.7)	142 (57.3)	0.92 (0.79–1.08)	0.31	1.15 (0.99–1.34)	0.07
Missing	204 (47.1)	142 (57.3)	1.02 (0.90–1.15)	0.80	1.24 (1.00–1.55)	0.05
**Current substance abuse**						
No	528 (46.9)	597 (53.1)	1.00	-	1.00	-
Yes	26 (29.5)	62 (70.5)	0.63 (0.45–0.87)	0.006	0.70 (0.51–0.96)	0.03
Missing	212 (47.0)	239 (53.0)	1.00 (0.89–1.12)	0.98	0.93 (0.73–1.19)	0.55
**Current depression**						
No	489 (45.5)	586 (54.5)	1.00	-	-	-
Yes	94 (44.5)	117 (55.5)	0.98 (0.83–1.15)	0.80	-	-
Missing	183 (48.4	195 (51.6)	1.06 (0.94–1.20)	0.32	-	-

^a^Row percents

^b^Median with first and third quartiles

^c^The multivariable model included clinically relevant variables selected *a priori* (age, sex, race ethnicity, BMI, chronic kidney disease, diabetes mellitus, smoking, alcohol abuse, ART duration) in combination with additional variables demonstrating a moderate statistical association (p<0.80 or >1.20) with hypertension in univariate analyses (insurance status, CD4, HIV viral load).

^d^Per each 5 year increase in ART duration, 1 year increase in time under care at the clinic, and 1 visit increase in visit frequency.

Abbreviations: ART, antiretroviral therapy; BMI, body mass index; CI, confidence interval; CVD, cardiovascular disease; HIV, human immunodeficiency virus; IQR, interquartile range; IVDU, intravenous drug use; MSM, men who have sex with men; PR, prevalence ratio (log binomial regression); UAB, University of Alabama at Birmingham

Missing data: HIV transmission risk factor, 30 (plus 1 patient excluded for risk factor of hemophilia/blood transfusion); chronic kidney disease, 11; CD4 cell count, 7; VL, 10.

### Patient characteristics associated with controlled BP

The prevalence of BP control among patients treated for hypertension was lower among African-American patients compared with whites in univariate analysis (PR = 0.83, 95% CI: 0.72–0.95) and after multivariable adjustment for age, sex, BMI, CKD, DM, smoking, alcohol use, duration of antihypertensive medication use, and antihypertensive regimen intensity and frequency (PR = 0.80, 95% CI 0.69–0.93, Table 3). Duration of antihypertensive medication use ≥5 years was associated with higher prevalence of BP control when compared with duration of antihypertensive medication use <1 year (PR-1.60, 95% CI: 1.00–2.56).

**Table 3:** Characteristics associated with blood pressure control among 699 HIV-infected patients on antihypertensive medication attending the UAB 1917 HIV Clinic in 2013.

Characteristic	BP controlled	BP not controlled	Univariate analysis		Multivariable analysis^c^	
	(n = 380)	(n = 319)				
	n (%)^a^ or	n (%)^a^ or	Prevalence ratio	p-value	Prevalence ratio	p-value
	median (IQR)^b^	median (IQR)^b^	(95%CI)		(95%CI)	
**Age, years**						
<40	35 (47.3)	39 (52.7)	1.00	-	1.00	-
40–59	278 (55.8)	220 (44.2)	1.18 (0.92–1.52)	0.20	1.12 (0.87–1.44)	0.39
≥60	67 (52.8)	60 (47.2)	1.12 (0.83–1.49)	0.46	1.07 (0.79–1.46)	0.65
**Sex**						
Female	104 (56.5)	80 (43.5)	1.00	-	1.00	-
Male	276 (53.6)	239 (46.4)	0.95 (0.82–1.10)	0.49	0.87 (0.73–1.02)	0.09
**Race**						
White	183 (60.2)	121 (39.8)	1.00	-	1.00	-
African American	197 (49.9)	198 (50.1)	0.83 (0.72–0.95)	0.006	0.80 (0.69–0.93)	0.004
**HIV transmission risk factor**						
Heterosexual	141 (50.9)	136 (49.1)	1.00	-	-	-
MSM	205 (58.4)	146 (41.6)	1.15 (0.99–1.33)	0.06	-	-
IVDU	31 (50.8)	30 (49.2)	1.00 (0.76–1.31)	0.99	-	-
**Insurance status**						
Private	163 (53.4)	142 (46.6)	1.00	-	-	-
Public	143 (56.3)	111 (43.7)	1.05 (0.91–1.22)	0.50	-	-
None	74 (52.9)	66 (47.1)	0.99 (0.82–1.19)	0.91	-	-
**BMI, kg/m**^**2**^						
Normal/Underweight (<25)	118 (55.9)	93 (44.1)	1.00	-	1.00	-
Overweight (25–29.9)	129 (55.4)	104 (44.6)	0.99 (0.84–1.17)	0.91	1.03 (0.88–1.22	0.69
Obese (≥30)	133 (52.2)	122 (47.8)	0.93 (0.79–1.10)	0.42	0.97 (0.81–1.16)	0.70
**Chronic kidney disease**						
No	314 (54.8)	259 (45.2)	1.00	-	1.00	-
Yes	64 (52.5)	58 (47.5)	0.95 (0.80–1.15)	0.64	0.99 (0.82–1.20)	0.89
**Diabetes mellitus**						
No	317 (54.5)	265 (45.5)	1.00	-	1.00	-
Yes	63 (53.9)	54 (46.1)	0.99 (0.82–1.19)	0.88	0.94 (0.78–1.14)	0.54
**History of CVD**						
No	331 (53.9)	283 (46.1)	1.00	-	-	-
Yes	49 (57.7)	36 (42.3)	1.07 (0.88–1.30)	0.50	-	-
**Duration of ART, years**	8.3 (4.3–12.9)	8.5 (4.3–13.2)	1.00 (0.94–1.06)^d^	0.96	**-**	**-**
**CD4 count, cells/μL**						
≥200	355 (54.0)	302 (46.0)	1.00	-	-	-
<200	24 (60.0)	16 (40.0)	1.11 (0.85–1.44)	0.43	-	-
**HIV viral load, copies/mL**						
≥200	32 (55.2)	26 (44.8)	1.00	-	-	-
<200	346 (54.3)	291 (45.7)	0.98 (0.77–1.25)	0.90	-	-
**Time in care at HIV clinic, years**	8.7 (4.3–13.7)	8.2 (4.5–13.5)	1.00 (0.99–1.02)^d^	0.87	-	-
**Cumulative visits in last 12 months**	3 (2–4)	3 (2–4)	0.99 (0.94–1.05)^d^	0.75	-	-
**Adherent to ART**						
No	51 (52.6)	46 (47.4)	1.00	-	-	-
Yes	211 (53.7)	182 (46.3)	1.01 (0.83–1.26)	0.85	-	-
Missing	118 (56.5)	91 (43.5)	1.07 (0.86–1.34)	0.53	-	-
**Current smoking**						
No	203 (54.1)	172 (45.9)	1.00	-	1.00	-
Yes	77 (52.7)	69 (47.3)	0.97 (0.81–1.17)	0.78	1.01 (0.86–1.18)	0.93
Missing	100 (56.2)	78 (43.8)	1.04 (0.89–1.22)	0.65	1.03 (0.80–1.31)	0.84
**At risk alcohol use**						
No	233 (55.2)	189 (44.8)	1.00	-	1.00	-
Yes	46 (50.0)	46 (50.0)	0.91 (0.73–1.13)	0.38	0.88 (0.70–1.10)	0.26
Missing	101 (55.6)	84 (45.4)	0.99 (0.85–1.16)	0.89	0.97 (0.80–1.16)	0.72
**Current substance abuse**						
No	255 (52.8)	228 (47.2)	1.00	-	-	-
Yes	14 (60.9)	9 (39.1)	1.15 (0.82–1.62)	0.41	-	-
Missing	111 (57.5)	82 (42.5)	1.09 (0.94–1.26)	0.26	-	-
**Current depression**						
No	243 (54.1)	206 (45.9)	1.00	-	-	-
Yes	45 (52.9)	40 (47.1)	0.98 (0.79–1.22)	0.84	-	-
Missing	92 (55.8)	73 (44.2)	1.03 (0.99–1.21)	0.72	-	-
**Duration of anti-hypertensive medication use**						
<1 year	13 (37.1)	22 (62.9)	1.00		1.00	
1 - <5 years	168 (54.7)	139 (45.3)	1.47 (0.94–2.29)	0.09	1.55 (0.97–2.47)	0.07
≥5 years	199 (52.4)	158 (44.3)	1.50 (0.97–2.33)	0.07	1.60 (1.00–2.56)	0.05
**Regimen intensity (number of antihypertensive drug classes)**						
One class	151 (53.5)	131 (46.5)	1.00	-	1.00	-
Two classes	147 (58.1)	106 (41.9)	1.09 (0.93–1.26)	0.29	1.08 (0.93–1.26)	0.31
Three or more classes	82 (50.0)	82 (50.0)	0.93 (0.77–1.13)	0.47	0.98 (0.80–1.20)	0.87
**Regimen frequency**						
Once daily	262 (56.5)	202 (43.5)	1.00	-	1.00	-
More than once daily	118 (50.2)	117 (49.8)	0.89 (0.77–1.03)	0.13	0.88 (0.75–1.03)	0.12

^a^Row percents

^b^Median with first and third quartiles

^c^The multivariable model included clinically relevant variables selected *a priori* (age, sex, race ethnicity, BMI, chronic kidney disease, diabetes mellitus, smoking, alcohol abuse, antihypertensive medication duration, regimen intensity and frequency). We allowed for selection of additional variables based on a moderate statistical association with BP control in univariate analysis, but none qualified.

^d^Per each 5 year increase in ART duration, 1 year increase in time under care at the clinic, and 1 visit increase in visit frequency.

Abbreviations: ART, antiretroviral therapy; BMI, body mass index; CI, confidence interval; CVD, cardiovascular disease; HIV, human immunodeficiency virus; IQR, interquartile range; IVDU, intravenous drug use; MSM, men who have sex with men; PR, prevalence ratio (log binomial regression); UAB, University of Alabama at Birmingham

Missing data: HIV transmission risk factor, 30 (plus 1 patient excluded for risk factor of hemophilia/blood transfusion); chronic kidney disease, 11; CD4 cell count, 7; VL, 10.

## Discussion

We found that African Americans had a higher prevalence of hypertension and lower prevalence of achieving BP control (SBP/DBP <140/90 mmHg) on antihypertensive treatment compared with whites among a southeastern US HIV cohort. Beyond racial disparities, findings related to the overall prevalence and control of hypertension in our cohort are also concerning. Nearly half of the patients with HIV in the current study had hypertension, and despite a high prevalence of antihypertensive treatment, only half of treated patients achieved BP control.

The 10% absolute difference in prevalence of BP control observed in HIV-infected African Americans in our study is clinically relevant and has potential bearing on population health if similar differences exist across the US. In the Swiss HIV Cohort Study, each 10 mmHg higher SBP was associated with an 18% increased risk for cardiovascular events [8]. Our finding that African Americans were also more likely to have SBP/DBP in the range ≥ 160/100 mmHg further underscores the potential clinical impact of the racial disparity in BP control among HIV-infected patients on rates of CVD and CKD, and outcomes related to these conditions. In one large HIV cohort study of patients on ART, African-American men experienced a 7.2% larger standardized 10-year all-cause mortality risk compared to white men, and African-American women a 7.9% larger risk, after controlling for pre-ART HIV-related health status [30]. This excess mortality is greater than the disparity observed between African-Americans and whites in the general population. While the reasons behind this are unclear, it is possible that a synergistic effect of disparities in post-ART HIV-related factors and non-HIV-related comorbidities such as hypertension, CVD, and CKD contributes.

The higher observed prevalence of hypertension among HIV-infected African Americans compared with whites is consistent with findings from general population studies and other HIV cohorts in the US, and remains incompletely understood [15–17, 31, 32]. Similar to other studies, we observed that this disparity in hypertension prevalence remains even after adjustment for sociodemographic characteristics and traditional risk factors [20, 21]. Although data are limited, biologic and genetic factors may contribute [31, 33]. However, there is considerably higher prevalence of hypertension among African Americans than persons of African origin in other geographic regions, suggesting cultural and environmental factors may play an important role [34, 35]. As with the general population, further studies among HIV-infected patients with hypertension are needed on the impact of factors traditionally not captured in cohort studies such as diet, chronic stress, neighborhood conditions, and racial discrimination [36–39]. HIV-related stigma has been correlated with poor physical health, but to our knowledge no studies have examined the impact of HIV-related stigma on hypertension and BP control [40]. A better understanding of the mechanisms underlying the high prevalence of hypertension among African Americans with HIV is needed for the development of preventive interventions.

In our study, African-American race was associated with lower prevalence of BP control among patients treated with antihypertensive medication; a similar racial disparity in BP control has been noted in the general US population [19, 20]. The disparity in BP control among our African-American patients persisted even after accounting for traditional risk factors for hypertension and antihypertensive regimen intensity, which has also been observed in the general population [32, 41]. There are limited data regarding factors which account for the lower prevalence of BP control in African Americans. Studies in the general population have reported associations with antihypertensive medication adherence, BP-related beliefs/attitudes, and health literacy [42–44]. These factors represent potential modifiable intervention targets to improve BP control among African Americans, and are worthy of further investigation among persons living with HIV infection.

Our findings are consistent with a recent study conducted among >24,000 HIV-infected patients in the Veterans Health Administration, which found racial disparities in control of HIV, blood pressure, and diabetes [45]. In addition to traditional risk factors for hypertension, this study was able to control for geographic region and neighborhood social disadvantage. However, it did not include variables related to antihypertensive medication use and evaluated a predominantly male population. While 92% of our HIV-infected patients on ART had controlled HIV (VL <200 copies/mL), only 54% of patients on antihypertensive medications had controlled BP. A fundamental difference between treating HIV and hypertension is that ART doesn’t require regimen intensification to achieve viral suppression, whereas titration of antihypertensive medications is often needed to achieve BP control. To our knowledge there are no published studies evaluating clinical inertia (failure of providers to titrate antihypertensive regimen) in response to uncontrolled BP among HIV-infected patients. We have previously observed clinical inertia related to lipid management in our HIV cohort [46].

Further, little is known about whether HIV-infected patients are more likely to be adherent to ART than medications for other chronic conditions and whether racial disparities in adherence to non-ART medications exist. There has been at least one study that compared adherence to ART versus antihypertensive medications [47]. Adherence to ART was better, but the difference was small (proportion of days covered 85% versus 83%, p = 0.013) and of uncertain clinical significance. Although a high proportion of our patients on ART had suppressed VL, African Americans had lower prevalence of ART adherence and viral suppression than whites. It’s possible that African Americans in our cohort also have lower adherence to antihypertensive medications, as has been observed in studies in the general population [42, 43].

Our study has known and potential limitations. Results from a single university-based clinic in the southeastern US may not be generalizable to other HIV clinical settings or geographic regions. Our findings may be affected by survival or selection bias, as patients who died prior to December 31, 2013 or who were lost to follow-up prior to 2013 were not included. However, our findings of higher prevalence of hypertension and lower prevalence of blood control among HIV-infected African Americans are consistent with prior studies in the general population and in HIV cohorts. Our study is focused on patients engaged in routine care in the current era and results may not be generalizable to HIV-infected patients who are not engaged in care.

There are likely unmeasured confounders for which we have not accounted. For instance, we lacked data on some factors which potentially affect development and/or control of hypertension, such as salt and potassium intake, physical activity, waist circumference, and lipodystrophy, as these are not routinely captured in our HIV care clinic [7, 43, 48–50]. Pharmacy refill data are not available in our cohort and thus we could not assess the impact of adherence on blood pressure control. However, we note our ability to control for several important clinical and behavioral factors available through our EHR and PRO platforms. As this was a secondary data analysis, we were unable to use a standardized protocol for BP measurement and had to rely on measurement obtained during routine clinical care to assess control. However, we note that routine clinic BPs are reflective of how BP control is assessed and informs provider treatment decisions in real-world settings.

In conclusion, we observed a higher prevalence of hypertension and lower prevalence of BP control among African-American HIV-infected patients, even when adjusting for traditional risk factors and antihypertensive regimen intensity. Notably, as observed for ART, no racial differences in prevalence of antihypertensive treatment were observed, but suboptimal control of BP among African Americans was seen, analogous to lower frequencies of viral suppression. Future studies on racial disparities in hypertension among HIV-infected patients should incorporate measures related to chronic stress, racial discrimination, and health beliefs, and also antihypertensive medication adherence, as potential mediators of the association between race and BP control, as has been evaluated for other populations. Such studies are needed to inform interventions for the prevention and control of hypertension in African Americans with HIV in order that this population may more fully realize the benefits of ART and HIV care.

## Supporting information

S1 Dataset (XLSX)

S2 Dataset (XLSX)
